# Infrastructure for Integration of Legacy Electrical Equipment into a Smart-Grid Using Wireless Sensor Networks

**DOI:** 10.3390/s18051312

**Published:** 2018-04-24

**Authors:** Paulo Régis C. de Araújo, Raimir Holanda Filho, Joel J. P. C. Rodrigues, João P. C. M. Oliveira, Stephanie A. Braga

**Affiliations:** 1 Federal Institute of Ceará (IFCE), 60040-215 Fortaleza, Brazil; pauloregi@gmail.com (P.R.C.d.A.), pregis@ifce.edu.br (J.P.C.M.O.); stephanie.abraga@gmail.com (S.A.B.); 2 PPGIA, University of Fortaleza (UNIFOR), 60811-905 Fortaleza, Brazil; raimir@unifor.br; 3 National Institute of Telecommunications (INATEL), 37540-000 Santa Rita do Sapucaí, Brazil; 4 Instituto de Telecomunicações, 1049-001 Lisbon, Portugal; 5 ITMO University, 197101 Saint Petersburg, Russia

**Keywords:** wireless sensor network, WSNS, smart grid, smart metering, middleware, real deployment

## Abstract

At present, the standardisation of electrical equipment communications is on the rise. In particular, manufacturers are releasing equipment for the smart grid endowed with communication protocols such as DNP3, IEC 61850, and MODBUS. However, there are legacy equipment operating in the electricity distribution network that cannot communicate using any of these protocols. Thus, we propose an infrastructure to allow the integration of legacy electrical equipment to smart grids by using wireless sensor networks (WSNs). In this infrastructure, each legacy electrical device is connected to a sensor node, and the sink node runs a middleware that enables the integration of this device into a smart grid based on suitable communication protocols. This middleware performs tasks such as the translation of messages between the power substation control centre (PSCC) and electrical equipment in the smart grid. Moreover, the infrastructure satisfies certain requirements for communication between the electrical equipment and the PSCC, such as enhanced security, short response time, and automatic configuration. The paper’s contributions include a solution that enables electrical companies to integrate their legacy equipment into smart-grid networks relying on any of the above mentioned communication protocols. This integration will reduce the costs related to the modernisation of power substations.

## 1. Introduction

Electricity generation, transmission, and distribution are essential for the development of modern society. Their underlying monitoring and control activities are performed by a variety of electrical system components. Current trends aims towards the automated operation of these components, particularly devices that are known as intelligent electronic devices (IEDs). The interconnection, communication, and control of these devices are embodied by the concept of smart grid, which is explained in [1,2,3,4,5].

In addition, another trend aims to monitor electrical equipment that is installed outside the power substation, at electricity distribution or transmission lines, or even at the point of consumption. However, the distance from the substation makes these monitoring tasks difficult and costly, provided that the communication between electrical equipment and the power substation relies on wired networks (e.g., Ethernet). Hence, the best option is to use wireless communication. In fact, the low cost and energy consumption of radio-frequency (RF) communication is more convenient, thus making wireless sensor networks (WSNs) an excellent solution for monitoring tasks over wide areas. To this end, electrical equipment can be connected to a sensor node and communicate with the power substation control centre (PSCC) through WSNs.

A typical PSCC requires information from different systems and controls several IEDs installed in the electricity distribution network. The wide variety of protocols, interfaces, and proprietary systems to manage the required communication and automation infrastructure represents the major barrier to the effective deployment of smart grids. To overcome standardisation issues in this area, various protocols were developed, such as the distributed network protocol 3 (DNP3) [6], IEC 61850, and Modbus [7]. Likewise, a WSN for integrating electrical equipment into a smart grid should use standardised communication protocols at its sensor nodes to interact with the components or control system of the smart grid.

The correct operation of electrical systems is critical, because faults might cause power outages affecting neighbourhoods or even entire cities, with the consequences impacting society as a whole. Thus, systems supporting and operating smart grids should meet certain important requirements, such as short response time and communications security. Hence, the same requirements should be met by communication protocols, for WSNs enabling the smart grid to comply with suitable response time and communications security, and to improve the operation of every system composing the smart grid.

To successfully integrate legacy electrical equipment into a smart grid that uses standard communication protocols (e.g., DNP3, IEC 61850, and Modbus), we propose a suitable infrastructure. The infrastructure consists of a ZigBee-based WSN that operates under the IEEE 802.15.4 standard, where the sensor nodes are connected to the legacy equipment, which then communicates with the PSCC through the sink node. The sink node, which has a wired connection (e.g., via Ethernet) to the central controller, runs a middleware that uses the Internet Protocol (IP) for communication between the sensor nodes and a terminal installed in the PSCC. In particular, the middleware mainly translates messages communicated between the PSCC and the electrical equipment in the smart grid. Furthermore, the proposed infrastructure allows guaranteeing the above mentioned requirements for smart-grid applications, including short response time, communications security, and even automatic configuration capabilities. To test the infrastructure, we setup an scenario to perform electrical measurements, where we evaluated the features and requirements of a power metering application.

The main difference between the middleware-based infrastructure proposed in this study and other available middleware-based solutions is the possibility of integration using the following smart-grid protocols: IEC 61850, DNP3, and Modbus. Many solutions do not use standard communication protocols for smart grids, and other systems and architectures communicate using only the IEC 61850 protocol, such as approaches presented in Section 2. In addition, similar solutions meet the latency requirements for smart-grid applications [8], but none complies with all the characteristics presented in this study. Then, the main contributions of this paper are the following:

- This solution enables electrical companies to integrate their legacy equipment into smart-grid distribution networks using the following smart-grid protocols: IEC 61850, DNP3, and Modbus.
- The infrastructure proposed in this study provides the facilities to guarantee the important requirements for smart-grid applications, such as short response time, communications security, and even automatic configuration capabilities.
- The infrastructure presented in this paper allows both the interoperability of electrical equipment from different manufacturers and the reduction of costs related to the modernisation of power substations.

The rest of the paper is organised as follows. Related work is presented in Section 2. In Section 3, the materials and methods used in this study are detailed. In Section 4, we describe the experiments and discuss their results. Finally, in Section 5, we conclude the paper and present future research lines.

## 2. Related Work

WSNs have been used to improve different aspects related to smart-grid operation, such as bidirectional communication and delivery, real-time seamless monitoring, energy quality and efficiency, and reliable and low-cost solutions for control management [9,10,11]. However, the integration of WSNs into the smart-grid depends on supporting technologies, such as middleware, frameworks, or infrastructures. For instance, Abid et al. [12] proposed a wireless architecture for a local advanced metering infrastructure using wireless mesh networks, and a middleware to manage the configuration of its different components. In [8], Shi et al. described a communication platform based on a data distribution service for smart micro-grids, which consists in a real-time monitoring system with a supporting middleware for communication over an IP network. Unlike the approach presented in this paper, this solution does not allow electrical equipment communicate using smart grid protocols. Zhou and Joel [13] described an integrated middleware for heterogeneous services related to smart grids. Specifically, they propose a service-oriented middleware that extends the system monitoring and control capabilities to the end user. Differently from the middleware presented in our study, the solution discussed by the authors does not satisfy important requirements of smart grid, such as response time, scalability, and communication security. In [14], Zhou and Joel proposed a quality-of-experience framework to allow power scheduling in smart grids. Likewise, Zaballos et al. [15] detailed a framework based on a ubiquitous sensor network with a decentralized middleware to coordinate the smart-grid operation and achieve end-to-end integration of heterogeneous technologies. This work focuses on the heterogeneity of technologies, but does not offer any solution to communicate legacy equipment to a smart grid. In addition to managing heterogeneity, the middleware proposed in this paper also enables the integration of equipment without standardized communication. A middleware for smart microgrids is presented in [16], and it acts as interface between users in a smart building and an advanced network infrastructure, thus providing functionalities related to data exchange in microgrids and support to hardware heterogeneity. Wilcox et al. [17] proposed a middleware, called Director, that operates between the running applications and network interfaces, and allows communication using a message-based protocol. Director mainly commands the network/socket application programming interfaces to generate and select the most suitable interfaces depending on the application requirements. In [18], Ibrahim et al. demonstrated a generic architecture for demand response programs in a smart grid. The architecture is divided into four concentric layers, namely context, operation, infrastructure, and platform, which are related to technical, environmental, and economic aspects to improve energy efficiency.

A novel technological paradigm has been proposed in [19]. According to the authors, this paradigm, called Fog of Everything (FoE), integrates the concepts of fog computing (FC) and the internet of everything (IoE). They also presented a small-scale of FoE prototype and its simulated energy and delay-efficient performance. In [20], Naranjo et al. presented a multi-tiered smart city architecture that utilizes fog computing (FC) for the devices. They developed a resource allocation model based on the fog computing that addresses the following components: device-to-device, device-to-fog node, and fog node-to-fog node. Pooranian et al. [21] proposed a stochastic method to solve microgrid operation problems. They proposed connecting the main grid and the microgrids to build multi-microgrids. With this topology, the energy can be supplied or absorbed, by either the microgrid or main grid. Based on this scenario, the authors proposed analyzing optimal power dispatch problem as a multi-objective function and solve it using NSGA-II algorithm.

In [22], Schroder et al. explored Long Range Wide Area Network (LoRaWAN) technology and suggested that this technology is an alternative to the big scenario of IoT and smart grids. The authors also compared LoRaWAN with the RF Mesh technology, which is widely adopted for this application. Unlike the wireless technology used in our study, the LoRaWAN technology does not have sufficient bandwidth to satisfy the amount of information exchanged in a DLMS communication. In [23], the LoRa technology is investigated with the aim of implementing distributed measurement systems (DMSs). The authors also defined indicators for the time-related performance and evaluated the capability of low-cost transceiver to satisfy certain time constraints. Barriquello et al. [24] presented the feasibility of LPWANs as smart grid communication networks (SGCNs) for rural areas. They used simulations to evaluate the performance of LoRaWAN based on network performance metrics, such as range, coverage, latency and packet delivery ratio. They also commented on opportunities and challenges in this research area. In [25], Wibisono et al. analysed the techno economic smart meter 2 ways implementation at Bali region by using two methodologies: techno-economic method and cost–benefit analysis. The authors also presented a business model and a regulatory aspect analysis, and concluded that LoRaWAN technology is a good option that can be used today. Saravanan et al. [26] explored the Power Wide Area Network (LPWAN) technology called LoRa to integrate IoT devices installed on the rural area. They proposed this technology to create a smart water grid management system, in which the quality of water can be measured by generating real time data. In [27], Vigni et al. proposed a new low cost device for fault location in power distribution systems.They use LoRa network as a communication link to report the presence and location of a fault in distribution systems.

In [28], Lloret et al. proposed an integrated IoT architecture for smart meter networks that include electricity, water and gas smart meters. They address some important issues, such as the communication protocol, data formats, the data gathering, and big data, that enable the architecture to be used in smart cities. Lloret et al. [29] presented a group-based architecture and protocol to improve the energy distribution in smart grids. According to the authors, their approach is innovative because there is no protocol to manage power distribution in electrical systems. A ZigBee based optimal scheduling system was presented by the authors in [30]. The authors proposed a system to manage energy for home appliances by shifting the load to off-peak hours using dynamic load scheduling. They used LabVIEW to monitor and control home appliances, which were integrated with a ZigBee-based sensor network.

Salvadori et al. [31] described a smart grid infrastructure using a hybrid network architecture. They proposed a digital system based on hybrid network architecture (HNA), which consists of wired and wireless infrastructures, controller area network (CAN), and a power line communications (PLC). According to the authors, this system is intended to integrate smart sensors and communication systems for real-time monitoring and control in smart grids. Although the authors commented that the system performs real-time monitoring, no temporal analysis was performed to determine the response times of event monitoring and control. The system proposed by the authors communicates using only the Modbus protocol, unlike our approach that enables communication using the DNP3, IEC 61850 and Modbus protocols.

Despite the abundant research, to the best of our knowledge, this is the first middleware-based infrastructure that aims to integrate legacy equipment into a smart grid. Moreover, many solutions do not use standard communication protocols for smart grids. For instance, middlewares presented in [32,33,34,35] do not communicate using any of the following smart-grid protocols: IEC 61850, DNP3, and Modbus. The systems and architectures presented in [36,37] communicate using only the IEC 61850 protocol. None of them allows the integration of WSNs into smart grids supporting all of these protocols. In addition, similar solutions meet the latency and security requirements for smart-grid applications, but none complies with all the characteristics presented in this study.

## 3. Materials and Methods

In this section, we present the proposed infrastructure with its components and strategies. These components consist of software applications that we implemented to run at both power substation equipment and WSN nodes. An important component of the infrastructure is the middleware that we implemented to run on the WSN sink node. This node is located in the power substation, and it has an Ethernet wired connection with the PSCC. Thus, the proposed middleware can communicate, via wired connection, with the supervisory software (e.g., ScadaBR) that runs on the central controller (PSCC computer). In this proposal, the WSN sensor node is wire-connected to the electrical equipment that is installed outside the power substation (e.g., power meter of the customer). For instance, the supervisory software performs requests to the middleware, which communicates with sensor nodes to receive electrical measurements from electrical equipment that is located outside the power substation. We also proposed strategies to satisfy important requirements for smart-grid applications, such as high security and suitable response time. These strategies are transparent to the user, provided that they are embedded in software applications. These components and strategies are addressed in the sequel.

### 3.1. Power Substation and Infrastructure Components

**Figure 1:**
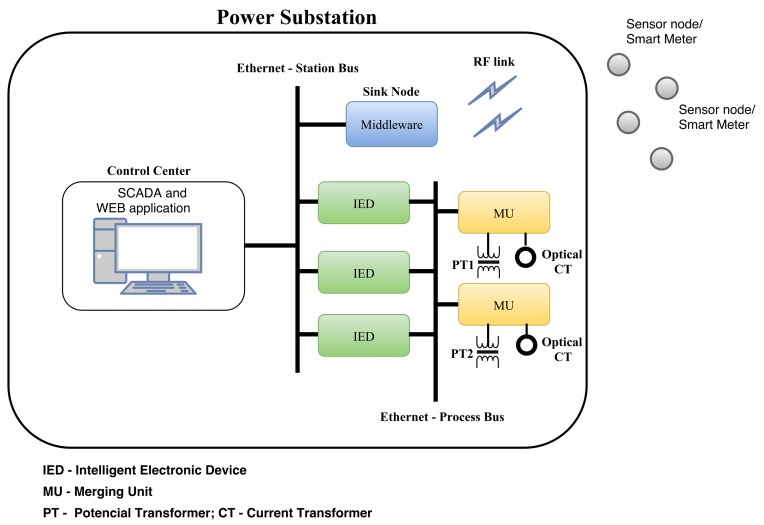
Power substation and proposed infrastructure.

The proposed infrastructure includes software applications and strategies that we implemented to run at the components of the power substation and at WSNs. Figure 1 shows the architecture of the power substation with the key components of the infrastructure installed on the central controller, sink, and sensor nodes. We considered that a supervisory control and data acquisition (SCADA) application [38] or the Distributed Test Manager (DTM) [39] are running on the central controller, the middleware is running on the sink node, and the strategies for complying with the smart-grid requirements have been implemented on both the sink and sensor nodes.

In addition, the PSCC and WSN sink node are located in the power substation, as shown in Figure 1. In contrast, the sensor nodes, which are connected to electrical equipment, can be installed outside the substation, for instance, in customer houses when the equipment is a power meter.

The PSCC, among other activities, monitors events and measurements of the electrical system and executes commands on the substation equipment. In addition, the control centre has a terminal executing SCADA to handle information from productive processes or physical installations. This terminal has a wired interfaced (e.g., Ethernet) with the WSN sink node.

We installed the proposed middleware in the sink node, which we considered to be a Raspberry PI model B [40], with the MRF24J40MC transceiver module [41]. We selected this hardware based on its processing power, storage capacity, and I/O capability, whereas the middleware consists of modules developed in Python. A similar research produced a middleware to be installed in the remote terminal unit (RTU), and its purpose was also to integrate electrical devices into a smart grid using a WSN [42]. However, old or small power substations do not have an RTU and this was the main motivation to develop a middleware for the same purpose but targeted to the sink node.

We implemented the sensor node, which can have a wired interface (using either the RS-485 or RS-232 standards) with electrical equipment, in the EK-TM4C123GXL ARM board (Texas Instruments Inc., TX, USA) [43]. The wired connection allows the sensor node either to receive measurements from the equipment, or to send commands to operate it. For instance, the sensor node can store data of recent measurements to answer requests of the sink node. The MRF24J40MC transceiver module of 2.4-GHz and IEEE 802.15.4 compliant [41] was also installed on the ARM board of the sensor node. This transceiver is a suitable wireless communication device for smart-grid applications, because it presents important features to improve reliability and security in communication, such as automatic acknowledgement response, frame check sequence (FCS) check, automatic packet retransmit capability, and hardware security engine (AES-128). In the electrical equipment, we decided to use a sensor node instead of only a wireless communication module, because we monitored other variables besides electrical magnitudes, such as temperature and humidity. In addition, remote functions such as equipment activation and deactivation can be implemented using a sensor/actuator node.

The RF communication between the sink and sensor nodes is based on the ZigBee specification, and sensors can be organised according to mesh or hierarchical topologies. ZigBee is a reliable, low power, low cost and efficient technology, and it is widely used in home area network (HAN). However, due to these characteristics, it is also used in smart-grid applications, as shown by the authors in [44,45,46,47]. In [44,48], the authors analysed and concluded that ZigBee is a suitable technology for smart-grid communication. In [49], a smart city applications is described. The authors used a ZigBee sensor network and worldwide interoperability for microwave access (WiMAX) to control an isle of street lighting. To solve the communication problem of smart-metering applications, the smart meters of a street must communicate with the nearest gateway (or cluster head) using ZigBee technology, and this gateway communicates with the middleware (sink node) using a long range wireless communication technology, such as general packet radio service (GPRS), long range wide area network (LoRaWAN), global system for mobile communications (GSM) or WiMAX.

The following reasons motivated us to use ZigBee technology in this study:

- The focus of this study is the smart metering concept, and leading smart-meter manufacturers, such as Lands+Gyr and Itron, use ZigBee technology or IEEE 802.15.4 standard for wireless communication of their smart meters [50,51].
- There are real applications using ZigBee technology for smart metering infrastructures. For instance, the gridstream smart metering solution from Landis+Gyr for Helen Electricity Network uses ZigBee technology [50]. Southern California Edison installed millionth Itron OpenWay Smart Meter that uses ZigBee technology for its communication [52]. In France, a first lot of 300,000 smart meters installed by Enedis Operator (Paris, France) communicate to IHDs using ZigBee or KNX interface [53]. More than 70 million ZigBee power meters have being deployed by dozens of utility companies in USA [54].
- Many devices for energy metering and management use ZigBee technology, such as universal controller ENOCEAN ZigBee (Schneider Electric, Inc. Rueil-Malmaison, Paris, France) [55], smart plugs (Develco, Inc. Aarhus, Denmark) [56], EMU-2 (Rainforest Automation, Inc. Burnaby, Canada) [57], OpenWay CENTRON smart meters (Itron, Inc. Liberty Lake, WA, USA) [58], and Landis+Gyr G370 (Landis+Gyr, Inc. Alpharetta, GA, USA) [59].
- There are smart metering technical specifications that suggested ZigBee technology as standard for HAN applications. For instance, the smart metering implementation programme (Department of Energy and Climate Change—UK Government) presented the smart metering equipment technical specifications—Version 2 (SMETS 2), which suggested the ZigBee technology as the standard for the HAN application layer [60].
- Power utilities in most regions of Brazil do not offer infrastructure (concentrating units, repeater units, and communication transformers) for PLC-based communication in their power grid. They do not authorize the use or installation of any equipment in their power network. Thus, no PLC-based communication test is possible.
- ZigBee technology has lower cost than other technologies such as LoRaWAN and Sigfox. These technologies require base stations or access points (APs) to operate, which must be installed on RF transmission towers. In many cases, RF transmission towers must be built, which increase the financial cost. DLMS is considered a standard protocol for smart metering, and there is no study or related work that evaluates whether the LoRa or Sigfox technology has sufficient bandwidth to satisfy the amount of information exchanged in a DLMS communication.

### 3.2. Infrastructure to Integrate Legacy Electrical Equipment to Smart Grid Using WSNs

As mentioned above, a wide variety of protocols, interfaces, and proprietary systems from different manufacturers are installed in electrical systems. Hence, the standardisation of these components is required. Furthermore, there are some communications requirements between electrical equipment and the PSCC, such as high security and suitable response time. These requirements, which enable operation and monitoring applications running in smart grids, are addressed in the next subsection.

#### 3.2.1. Integration Requirements

- Standardisation: Power substations are currently using standardised equipment for operation, monitoring, and communications. This facilitates the integration and interoperability among electrical devices, improving the power system maintenance and operation. In particular, communication protocols such as DNP3, IEC 61850, and Modbus are widely used in smart grids. Thus, a WSN-based integration of legacy electrical equipment and sensors into a smart grid requires the use of such standardised protocols. Moreover, a middleware must support the WSN sink node to enable the communication between different equipment and either the PSCC or another smart-grid system.
- Response Time: Smart-grid applications must comply with a suitable response time. In fact, the IEC 61850 protocol specifies a time constrain for message exchange. For instance, this protocol limits the transmission time of type 3 messages (i.e. low-speed messages) to 500 ms [61]. Likewise, we implemented a strategy to improve the response time for WSN communication, which has allowed to meet some time requirements of the IEC 61850 protocol. Specifically, the proposed strategy is based on two phases of the polling technique, which uses periodic requests at either the sink node or the device connected to the sensor node. We provide further details of this strategy in Section 3.3.2.
- Security: Security is a major concern in smart-grid applications. For instance, in a power metering application, the information of customer power consumption retrieved by the electricity utility must be treated with safety and reliability. Otherwise, a WSN transmitting information between the electricity utility and smart meters might be corrupted by a spy node, which could alter the consumption readings in the benefit of the customer. To avoid this type of problem, we propose the use of an encrypted channel with keys generated at the sink and sensor nodes to secure their communications. The keys are modified at runtime to hamper the inclusion of corrupted data, and we verified the effectivity of this encryption method, as shown in the corresponding results of Section 4.

#### 3.2.2. Integration Middleware

The proposed middleware shows a novel and different approach for the concentrator core which was developed in previous research [42]. However, as mentioned above, old or small power substations do not have an RTU, thus impeding the installation of the concentrator core middleware [42]. Consequently, the middleware proposed in this study is intended to run on the sink node and it presents different characteristics related to the previous middleware, such as integration in the outstation environment; wireless communication; possibility of long range communication using GPRS or GSM technologies in the sink node; communication of legacy equipment using the IEC 61850, DNP3 and Modbus protocols; focus on smart metering; and compliance with the response time requirements of the IEC 61850 standard. As shown in Figure 1, the sink node is located in the power substation, and it has an Ethernet wired connection with the PSCC using transmission control protocol (TCP)/IP.

**Figure 2:**
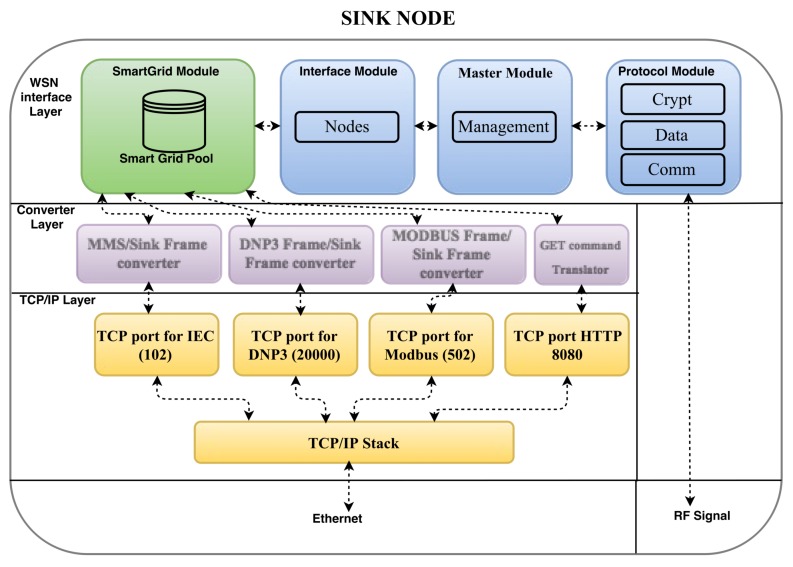
Middleware components within the sink node.

Figure 2 shows that the proposed middleware consists of Python modules that run at the converter and WSN interface layers. The functionality of these middleware modules are detailed as follows.

Master.py: This module commands the others and contains a Python class called “Management”, which has a method for initialising modules “protocol.py”, “smartgrid.py”, and “interface.py”. Other methods include “scannodes()”, “pingnodes()”, and “announce()”. Method “scannodes()” sends a broadcast message at different instants to discover new nodes in its coverage area; “pingnodes()” sends a message to each node in the routing and control table for checking the node status on the network, and it removes from the table the non-responding nodes; and, finally, “announce()”, which is called after “scannodes()”, searches for new nodes and tries to initialise node data, and it creates a new alias for the network interface, thus assigning a unique virtual IP address to a discovered sensor node. The IP address, ID, and basic information of the node are stored in the routing and control table. These methods correspond to the automatic configuration which allows a sensor to be seamlessly included in the network. Moreover, this automatic configuration might simplify scalability, which is out of the scope of this study. In addition, module “Master.py” contains a method called “request()” which starts any request on the WSN and aims to guarantee the security requirements for WSN communication, as detailed in Section 3.3.1.

Interface.py: This is another essential module in the middleware and contains a class called “nodes” with methods “request_lock()”, “request_release()”, “del_NODE()”, and “add_NODE()”. Methods “request_lock()” and “request_release()” operate as locks and prioritise the requests from the SCADA application to send data through the sensor network. Thus, these methods block any command sent from Web application, provided that the SCADA application is transmitting. In fact, for a smart-grid application, SCADA commands and requests must have top priority because it performs real-time and autonomous monitoring from the power substation. Still, the proposed middleware also allows requests to sensor nodes from a Web application, without the use of standard protocols for smart grids. Method “add_NODE()” is invoked by the above-mentioned method “announce()”, whereas method “del_NODE()” performs the reverse operation of “add_NODE()”, i.e., it removes the sensor node information from the routing and control table.

Protocol.py: This module basically specifies the WSN communication protocol. It contains the following classes: “crypt”, “data”, and “comm”. Class “crypt” performs data encryption and comprises methods “encrypt()”, “decrypt()”, “crypt()”, “setHASH()”, “getHASH()”, and “getNEXThash()”. Clearly, these methods allow encrypting and decrypting the data transmitted through the WSN, and create, configure, and modify the encryption keys. Hence, these methods are part of the security strategy for the sensor network communications. Then, class “data” basically consists of methods “encode()” and “decode()”, which convert the message format in the middleware to the request packet structure used by the WSN. Specifically, method “encode()” uses method “pack()” to construct the WSN request packet, and “decode()” uses method “unpack()” to extract information from the WSN responses. Finally, class “comm” is responsible for the communication between the Raspberry PI and the radio transceiver. It contains methods “_init_()”, “close()”, “cleanSerial()”, “send()”, and “receive()”, which set the transceiver parameters, close the serial connection, clean the serial buffer, send data, and receive data, respectively.

Smartgrid.py: This module creates a link between either the SCADA application or DTM and the sensor nodes, and contains a class called “smartpoll” that comprises methods “waitRequest()”, “sendResponse()”, and commInterface()”. Method “waitRequest()” waits for requests and receives them from the converter layer. Then, method “commInterface()” sends the request to module “Interface.py”. In addition, module “smartgrid.py” has a poll (buffer) to store the data (responses) received from the sensor nodes. These data can be used as responses for the SCADA application or DTM. Method “sendResponse()” sends responses stored in the poll to the converter layer.

ConverterLayer: The middleware is intended to receive requests from the supervisory control and data acquisition (SCADA) software and respond them with the values that are stored in the buffer area created by the servers of smart grid protocols. These servers represent the converter modules in the middleware converter layer. Thus, input signals can be represented by reading commands sent by the SCADA software, and output signals are the values returned as responses to these requests.

To receive requests from the TCP/IP layer, the modules in the converter layer create new servers on the ports related to protocols DNP3, IEC 61850, and Modbus. For instance, if the SCADA application performs a request using the Modbus protocol, the module for “Modbus frame/sink frame conversion” creates a new server at port 502 to receive the corresponding commands. Hence, the TCP port that receives the request determines the corresponding server for communication.

Module “DNP3conv.py” (DNP3 frame/sink frame converter) contains class “DNP3Server” with four methods: “startServer()”, “linkLayer()”, “transportLayer()”, and “AppLayer()”. Method “startServer()” instantiates the server and receives the request parameters from the SCADA application using the DNP3 protocol; “linkLayer()” extracts the fields from the DNP3 frame header and then reassembles the frame information to compose fragments; “transportLayer()” gathers and formats the fragments to compose the corresponding message. Method “AppLayer()” maps both the static data and point indices of the request into the electrical parameters recognised by the middleware and the sensor node IDs, respectively.

Likewise, module “MODBUSconv.py’ (Modbus frame/sink frame converter) contains five methods: “init()”, “setValues()”, “StartTcpServer()”, “getALLdata()”, and “getSINGLEdata()”. These methods perform several tasks, e.g., instantiate the server and receive the request parameters, extract the fields from the header of the Modbus application protocol, convert slave addresses into sensor node IDs, convert function code into request parameters, and construct the corresponding Modbus TCP messages.

Finally, module “IECconv.py” (manufacturing message specification (MMS) frame/sink frame converter) contains some classes, with the most important being “IedServer”, which has methods “IedServer_create()”, “IedServer_start()”, “IedServer_stop()”, and “IedServer_destroy()”, among others. These methods allow creating an IEC 61850 server application and manage both the MMS protocol stack and IEC 61850 data model. Moreover, they initialise the protocol stack, listen for client connections, provide process values to the MMS server, react to client events, stop the MMS server, and free all the resources allocated by the IedServer instance. The methods from class “IedServer” to provide process values (i.e. handling process values from a client application to the MMS server) include “IedServer_lockDataModel()”, “IedServer_updateAttributeValue()”, and ’IedServer_unlockDataModel()”.

Given that the converter layer of the proposed middleware must receive messages that comply with standard protocols for smart grids and translate them into a format recognised by the WSN, we proposed a memory mapping to facilitate this translation in previous research [62]. This mapping is based on an address relationship in the memory area, which we called the smart-grid poll, and further details can be found in [62]. Other smart-grid protocols can be easily adapted to the middleware. For instance, inter-control centre communications protocol (ICCP) communicates using the TCP/IP protocol, and uses the client/server model. This paradigm is used in the converter layer by the smart-grid protocol servers. Thus, the user can adapt an ICCP server to the converter layer so that an ICCP client can request information from the WSN. Moreover, the ICCP protocol communicates using MMS message protocol, which is already mapped by the middleware, and it is generally available as a native protocol embedded in some SCADA hosts.

### 3.3. Requirement Compliance for Smart-Grid Applications

#### 3.3.1. Communication Security in WSN

Although ZigBee specifies security management for communication, the security measures we propose can be used for communications in radio technologies that are not endowed with intrinsic security. In Section 3.2.1, we explain the importance of security in smart-grid applications. Specifically, we mention the WSN susceptibility to the spy node attacks, which turns measures such as encryption important to secure WSN communication. However, WSNs usually depend on sensor nodes implemented using microcontrollers with low processing power and storage capacity, thus severely restricting data encryption capabilities. Consequently, instead of using computationally expensive techniques such as public key steganography [63], advanced encryption standard [64], or secure hash algorithms [65], we can opt for encryption models with simpler algorithms and less complex computations. For instance, XOR encryption can be applied to the method known as one-time pad [66]. This encryption method requires a random key that has at least the same size of the information block to be encrypted, and it uses different keys to encrypt different information blocks, for the pair key/information block to never be repeated. In the sink node, an XOR operation is performed between the message and initial key “K0”. Then, this encrypted message and another key, “K1”, are sent from the sink node to the destination node. In the destination node, a new XOR operation is performed between the received message and key “K0” to obtain the original message. Likewise, the destination node encrypts the response with key “K1”, generates key “K2”, and sends the encrypted response and key “K2” to the sink node.

**Figure 3:**
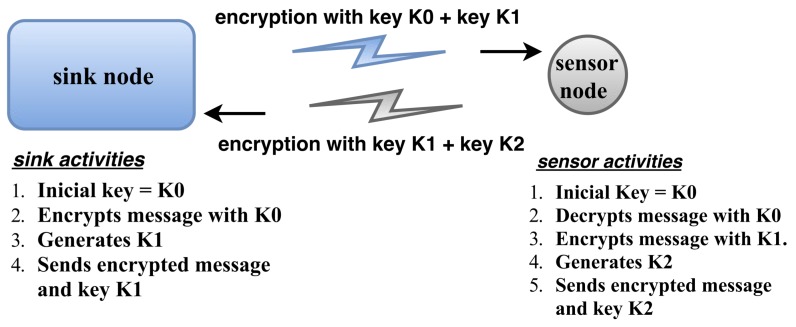
Encryption technique and activities in the sink and sensor nodes.

Thus, an encryption key is generated per sink-node request and per sensor-node response, as shown in Figure 3. The management of the keys is performed by the sink and the sensor nodes, and, whenever message re-routing occurs, the packet is retransmitted using the original key, for the receiver to successfully decrypt the entire message.

As shown in Figure 3, we employ a modified version of the Mersenne Twister, which is a widely used pseudorandom number generator [67], to create encryption keys. The seed of this modified version corresponds to the analogical output produced by an RF receiver connected to an input pin of the sink node. The receiver captures the RF signal from the environment, which is then amplified and applied to a digital/analogue converter. This analogue signal is converted into a digital value which corresponds to the seed of the encryption key generator. Finally, a simplified version of the Whirlpool hash [68] is used on the generated message, which is cut to the maximum desired size.

#### 3.3.2. Response Time in WSN Communication

**Figure 4:**
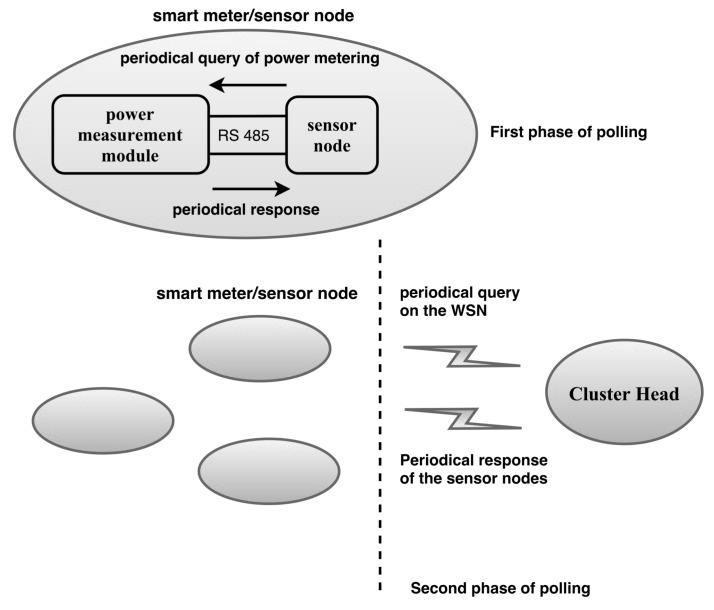
Diagram of WSN communication with two levels of polling.

To meet the response time requirements for smart-grid applications, we use strategies to reduce the communication time in WSNs. We consider two phases of the polling technique. In the first phase, which is illustrated at the left side of Figure 4, the sensor node periodically reads measurements from the electrical device. Thus, whenever the sink node requests information from the electrical device, updated values are available in the sensor node. This strategy has been proposed to minimise the communication time of some electrical equipment. For instance, the Cronos 7023 power meter (Eletra Energy, CE, Brazil) [69] has a communication time of approximately 1.1 s. In the second phase, provided that the WSN has a hierarchical topology, the cluster head (CH) periodically requests electrical measurements from each of the related sensor nodes and stores them for future requests from the sink node. For the first phase, we selected an interval to perform the periodic readings on the electrical equipment of 5 s, because some equipment presents communication times below that value. For the second phase, we selected an interval to perform the periodic requests to the sensor nodes of 10 s, which was based on a CH with 100 sensor nodes within its coverage area, and a packet transmission and reception time of 70 ms. In a hierarchical WSN, the CH can periodically receive and store the readings from its related sensor nodes, and respond to requests from the nodes when necessary. Thus, the time for a request from the sink node is reduced because additional requests to the nodes of the CH are not required. This approach is simpler than other techniques found in the literature. For instance, the authors in [70] proposed a real-time chain for transmitting the sensed data with delay reduction. According to the authors, this technique consists of broadcasting a message from grid head (GH) to grid head until all grid heads receive the message. Then, a real-time chain from a detected node to the sink is formed using any routing algorithm. They used simulation results to compare delays related to four scenarios. In [71], the authors presented two novel optimization models that find the optimum values of the end-to-end latency and power consumption in a clustered WSN. According to the authors, the optimization models are intended to evaluate latency requirements in smart-grid applications. They concluded that a clustered ZigBee-based WSN can guarantee some time constraints imposed by smart-grid protocols. This conclusion reinforces our approach, which uses a clustered ZigBee-based WSN with a polling technique to reduce the response time in WSN communication.

This strategy has allowed to meet one of the time requirements of the IEC 61850 protocol for critical operations in intra-substation environments. Other requirements such as those related with quality of service demand a high-speed message communication below 1 s [72]. As shown in Section 4, we obtained response times below 1 s in WSN communication.

#### 3.3.3. Automatic Configuration

We used two strategies to include new sensor nodes in the network without requiring manual configuration. The first strategy, which we proposed in [42], consists of an algorithm to include new sensors in a hierarchical WSN without manually configuring the sensors.

In this strategy, the inclusion process begins when a new sensor node is installed in the electrical system. The sensor node listens for messages from nearby CHs, and, for each received message, the node stores the radio signal strength, given by the received signal strength indicator. Then, it compares the signal strengths and selects the strongest CH. Finally, the node sends a message to the selected CH to inform the presence of a new sensor node, and the CH acknowledges this message by returning an ID to the sensor node. The ID is based on the corresponding table, which is stored in each CH. The CH also notifies the new sensor-node ID to the sink node in response to a request from method “scannodes()”, which is mentioned in the next paragraph.

The second strategy is based on module “Master.py” and comprises of two related methods, namely, “scannodes()” and “announce()”, which are detailed along with the “Master.py” description.

## 4. Experiments and Results

We performed experiments using a scenario with power meters connected to sensor nodes to allow remote readings of customer energy consumption. These experiments aimed to perform these readings using either a SCADA application or DTM. In the sequel, we describe the environmental setup.

### 4.1. Experimental Setup

We validated the proposed integration infrastructure in a laboratory environment. The test scenario consisted of a WSN with three sensor nodes and one sink node. As mentioned above, each sensor node, which is connected to a Cronos 7023 power meter, was implemented in a EK-TM4C123GXL ARM board equipped with a MRF24J40MC transceiver module. The sink node was implemented in a Raspberry PI model B and had an Ethernet connection with a computer, which was running the Windows 7 operating system, and the open-source licenses of ScadaBR and DTM.

**Figure 5:**
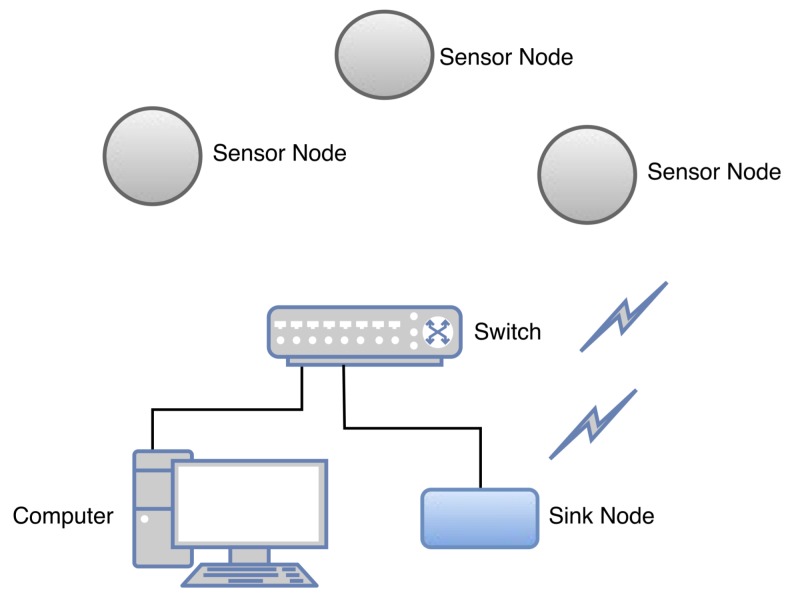
WSN topology and computer.

Figure 5 shows the WSN node distribution with a distance of 2 m between the sink and sensor nodes, for all the nodes to be within the same coverage area.

### 4.2. Results

After preparing the experimental setup, we performed a test to validate the middleware converter layer, i.e., to determine whether the requests made by the ScadaBR or DTM [39] using any of the smart-grid protocols (DNP3, IEC 61850, or Modbus) were fulfilled.

**Figure 6:**
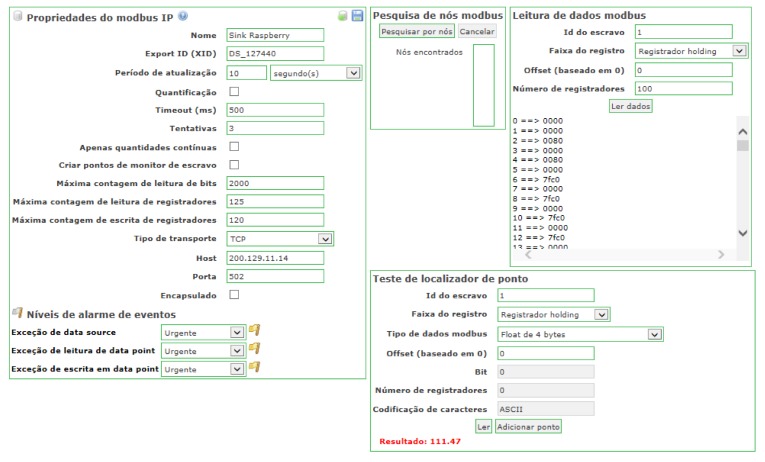
Screenshot of ScadaBR software using MODBUS protocol.

Figure 6 corresponds to a screenshot of ScadaBR, which is an open-source SCADA software. The screenshot shows a response to a request using the Modbus protocol. The left-side window details informations of the Modbus IP properties, such as the 10 s interval selected for reading updates, three retransmission attempts when a timeout expires, 500 ms timeout, TCP transport layer, port 502 for Modbus, and IP address 200.129.11.14 for the sink node. We assigned the TCP port number 502 as it is the port used for the Modbus protocol. The right-side window, called point locator test, shows information related to the characteristics of the point to be queried, such as Modbus data type (4 byte float), register type (holding register), number of registers (related to the data type), and character encoding. In addition, this window contains a command button called “read” to perform requests on a target sensor node (target point). Whenever activated, this button generates a request to the sensor node and retrieves the corresponding response (red colour string). Related to the middleware, the input signal is the request sent by the ScadaBR software, which is a read command to retrieve HR register contents stored in the Modbus stack. This stack is created in the middleware convert layer. From this experiment, we successfully retrieved a phase voltage of 111.47 V, which was stored in HR registers. This value, which is the output signal of the middleware, was measured by the power meter connected to the sensor node.

**Figure 7:**
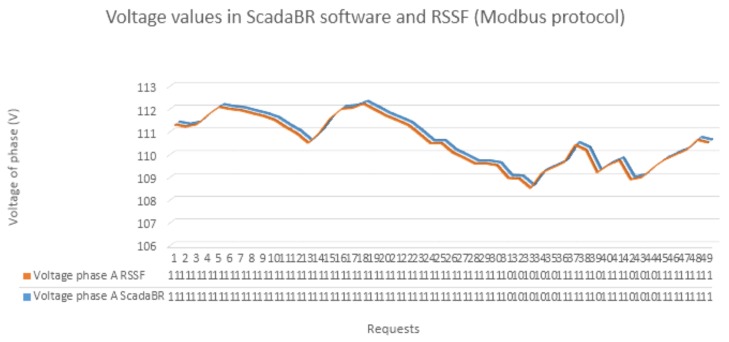
Comparison between the readings performed by ScadaBR and the sink node on the WSN.

In addition, we developed a Python program to present the voltage readings performed by the sink node directly on the sensor nodes. For each voltage-reading request from ScadaBR arriving at the sink node, the program records the corresponding response in an XML file, before the middleware sends the response to ScadaBR. The objective of this program was to compare the values received by ScadaBR with those obtained by the sink node from the sensor nodes. This comparison, which is illustrated in Figure 7, allowed verifying the accuracy of the converter layer when formatting voltage readings from the power meter into corresponding protocol data units.

**Figure 8:**
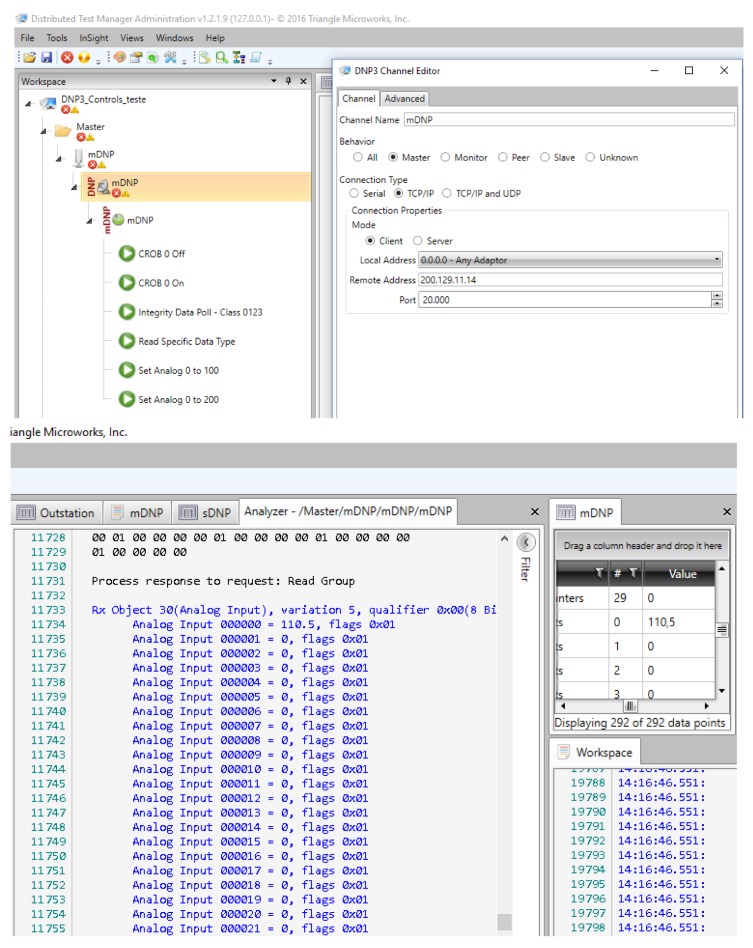
Screenshot of the DNP3 master configuration using DTM software.

The 3D graph in Figure 7 shows the outcome from 50 voltage readings from the sensor node 1 performed by both ScadaBR and the sink node. Their values are almost equal, showing only a negligible difference. Only few values exhibit differences in the order of hundredths or thousandths, given the rounding of ScadaBR. Similar results were obtained for sensor nodes 2 and 3. The top part of Figure 8 shows a screenshot of the DNP3 master configuration designed using a free version of DTM. We used DTM because ScadaBR does not implement the DNP3 protocol. The DNP3 channel editor is shown on the right side of the screenshot. The computer running DTM was configured as the master (behaviour), the connection was TCP/IP (connection type), the DTM request application was configured as client (mode), the remote address was the IP address of the sink node (200.129.11.14), and the port was 20,000, which is the standard TCP port for DNP3. The bottom part of Figure 8 shows the DNP3 response received by the DTM client. DNP3 requests performed by DTM software, which represent input signals for the middleware, are read commands to retrieve the fragments (DNP3 message structure) stored in the DNP3 stack. This stack is created by the DNP3 server in the middleware converter layer. In response, the middleware returns fragment objects stored in its DNP3 stack. The response at line 11,733 represents the parameters of the application response header, which has an object type of 30 (current value of the point), a point type corresponding to analogue input, and a variation of 5, which represents a floating-point value. Line 11,744 contains the voltage value of a power meter connected to a voltage stabiliser with a standard output of 110 V, which is the output value of the middleware.

**Figure 9:**
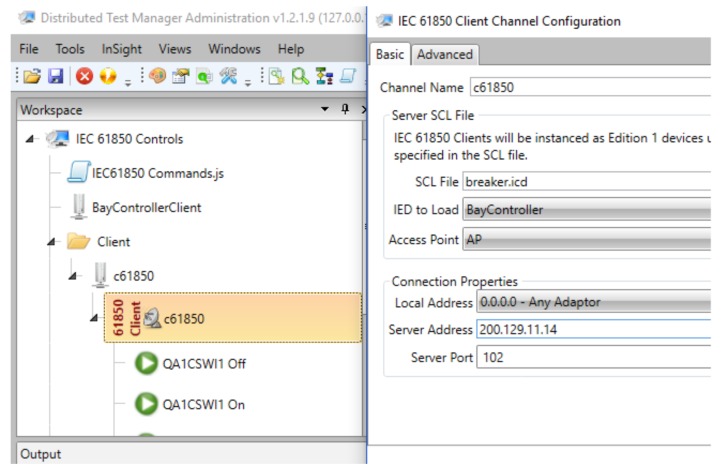
Screenshot of the IEC 61850 client configuration using DTM software.

Figure 9 shows a screenshot of the IEC 61850 client configuration, which was also designed using a free version of the DTM software, because ScadaBR does not implement this protocol either. The IEC 61850 channel editor is shown on the right side of the screenshot. In this case, the computer running DTM was configured as the client, the connection was TCP/IP, the server address was the IP address of the sink node (200.129.11.14), and we used port 102, which is the standard TCP port for the IEC 61850 service.

**Figure 10:**
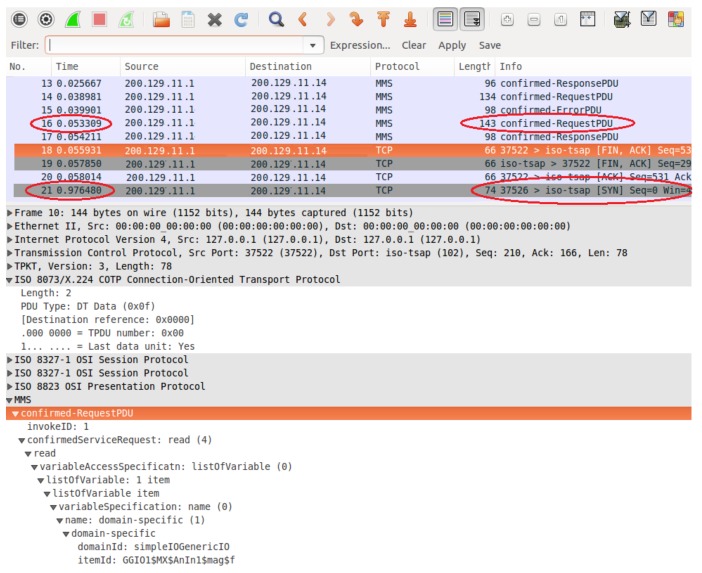
Screenshot of the confirmed IEC 61850 request captured by the pcap (packet capture) application.

**Figure 11:**
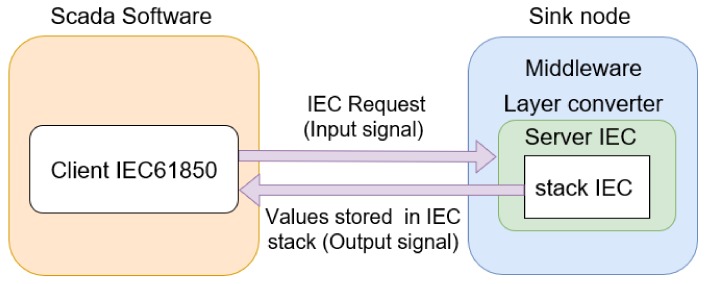
Inputs and outputs related to the middleware.

Figure 10 shows a screenshot confirming the DTM request, which was performed using the IEC 61850 protocol and retrieved using the pcap application for capturing packets. We selected this screenshot to clearly show both the service that was used in the request (read service) and the data object implemented for the measurement unit. Related to the middleware, input signals to test the IEC converter module are IEC requests performed by DTM software, which are read commands to retrieve data object attributes that represent phase voltages of the power meter. These object attributes, which represent the output signals of the middleware, are stored in the IEC 61850 stack (middleware converter layer). In Figure 11, a flow diagram represents input and output signals on an IEC 61850 communication between the SCADA software and the middleware. For instance, the input signal for the middleware is the IEC request sent by the SCADA software, and the output signals are the values stored in the IEC stack (middleware) which are returned to the SCADA software.

In Figure 10, the first orange row contains information such as the type of message protocol (MMS), the IP address of the destination node (200.129.11.14, the IP address of the sink node), and the message information (request confirmation). The IEC 61850 protocol uses the client-server model in its communication. Thus, the SCADA software runs the IEC 61850 client, which performs the requests and is always considered the source node. On the other hand, the sink node executes the IEC 61850 server, which must always respond to IEC client requests. The IEC server is always considered the destination node, since it is the destination of all SCADA software requests. Although the IEC 61850 server returns a response to the SCADA software, for the PCAP software, it is always identified as the destination node. Information on the source TCP port is shown on the line called “TransmissionControlProtocol”. This port was set to 102. In the IEC 61850 protocol, the measurement information from an electrical device is represented by a data object, and its structure is indicated by the “idemID” parameter of the “domain-specific” item. In “idemID”, expression “*f*” is the *type description* attribute, which is floating-point (i.e. voltage is represented by a floating-point value); “mag” is the *variable-name* attribute for the phase voltage magnitude; “AnIn1” represents the *named variable* object, which is related to the voltage value of the power meter connected to sensor node 1 (i.e., power meter voltage is an analogue input); “MX” is the *functional restriction* attribute which is related to measurement activities; and “GGIO” is related to the logical node and represents the virtual manufacturing device object.

The abovementioned results verify the applicability of the proposed middleware for communication between the WSN and a supervisory software running on a server which uses one of the three presented communication protocols for smart grids. Therefore, an electrical device connected to a sensor node can be integrated into a smart grid that supports the DNP3, IEC 61850, or Modbus protocols.

Next, we performed another test to evaluate the response time. The computer running DTM and the pcap application performed a series of requests to sensor node 1 (in our data model, AnIn1 is related to the power meter connected to sensor node 1) for retrieving the electrical voltage from the power meter. We configured DTM to use the IEC 61850 protocol, which presents tight constraints regarding response time. We used the pcap application to capture the transmitted and received data packets in the computer. Consequently, we could determine the time intervals between the requests and responses. Figure 10 shows a screenshot of the request/response procedure performed by DTM using the IEC 61850 protocol and captured by the pcap application.

**Figure 12:**
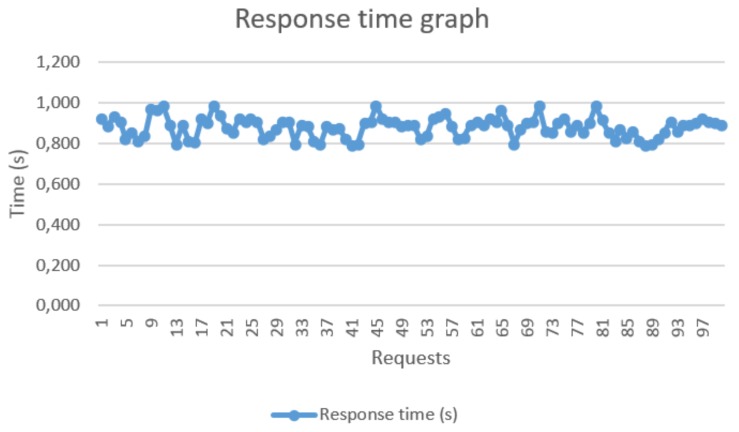
Records of the request/response times captured by pcap software.

The screenshot shows a sequence of packets, which contain the sequence number, event time, destination and source nodes, protocol type, and packet information. The two red marks in sequences 16 and 21 indicate the beginning of a request and its corresponding response. The instants when these events occurred were recorded: request began at 0.053 s, and its response reached at 0.976 s. Thus, we determined the response time to a request given by the difference between these instants. We performed 100 requests to sensor node 1 to evaluate the response time, which was determined from the difference between the instants of the request triggering and the response arrival. Figure 12 shows the corresponding response times with none of them exceeding 1 s. Thus, the proposed infrastructure satisfied the time requirements of the IEC 61850 protocol for critical operations in intra-substation environments. The suitable response time was achieved by using the polling technique, which allows the periodic reading of the measurements stored in the sensor node. In this case, the voltage value provided by the power meter was already stored in the sink node, as it performs periodic requests to the WSN sensor nodes.

**Figure 13:**
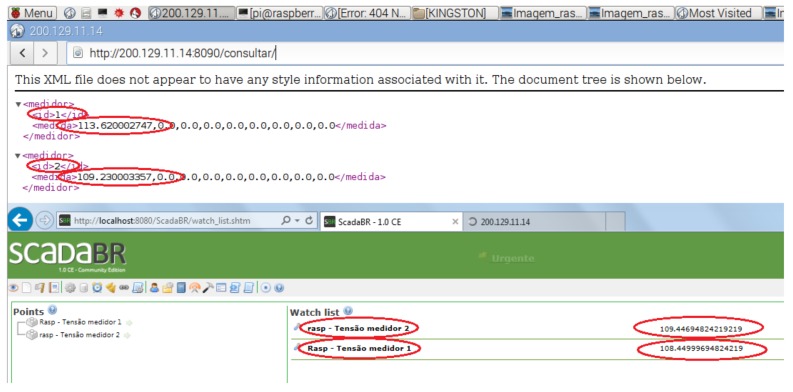
Screenshot of the responses captured by the Scadabr software and the local application with another sensor node inserted.

**Figure 14:**
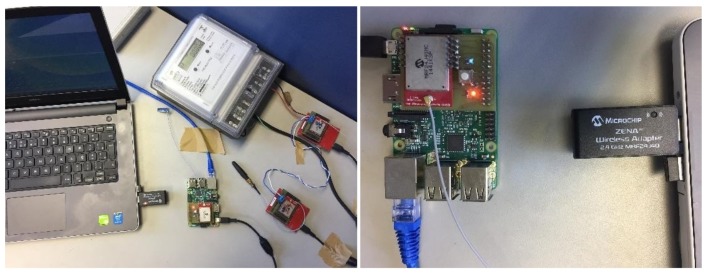
(**left**) WSN with a sniffer adapter to capture the packets, and (**right**) the sink node and the sniffer adapter.

Then, we setup a scenario to validate the automatic configuration of sensor nodes in the WSN. Initially, only one sensor node was installed and located near the sink node, which was running an application to perform requests on the nodes located within its coverage area. In this scenario, only one sensor node answered to each request. A similar procedure was performed using ScadaBR, but only named nodes were allowed to perform requests. We observed the same event, and verified that the unique sensor node answered to the request. Then, we installed and activated another sensor node, which was located near the sink node. We performed the same procedure, and requests were sent to the sensor nodes by both the sink-node application and ScadaBR. Figure 13 shows a screenshot with the responses that were obtained from the local application (top of the figure) and ScadaBR (bottom of the figure). Two responses from the local application can be observed, one related to sensor node 1, and the other related to sensor node 2. These sensors are shown in Figure 14. Likewise, the responses from ScadaBR also correspond to sensor nodes 1 and 2. The red marks in the figure represent the sensor node IDs and the electrical measurements. No configuration was performed on the sink node for the inclusion and communication with sensor node 2. This configuration was automatically performed by methods “scannodes()” and “announce()”, which are part of module “Master.py”. Thus, we verified the successful automatic configuration of new sensor nodes inserted in the WSN, which facilitates the network scalability.

**Figure 15:**
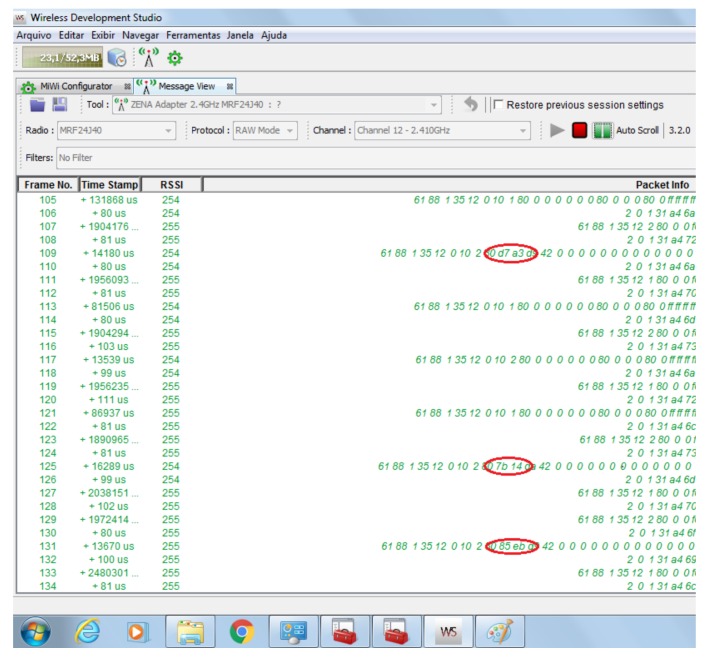
Screenshot of the data packets captured by the WDS software.

To verify the proposed security strategy, we setup another scenario considering the WSN communication. Figure 14 shows this scenario that consists of a WSN containing the sink node, two sensor nodes, and a spy node, which was connected to a notebook and supported by a ZENA wireless adapter (Microchip Technology Inc., Chandler, AZ, USA) [73]. This adapter is a protocol analyser for 2.4 GHz wireless communications and pre-programmed with a MiWi wireless protocol sniffer application. Thus, it can analyse the data packets that travel through the wireless network based on the ZigBee protocol and raw data. In this scenario, we performed a series of requests using ScadaBR and used the proposed security strategy to encrypt the message payload of the requests and responses. These encrypted messages travelled through the wireless network and were captured by the spy node. In fact, all the data packets in the WSN were captured by the spy node for one hour. Figure 15 shows a screenshot of the Wireless Development Studio, which is a Microchip software used to present the data packets captured by the ZENA wireless adapter.

**Figure 16:**
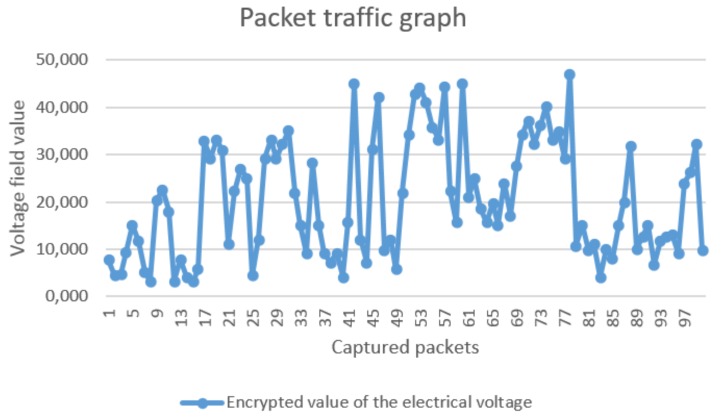
Screenshot of the encrypted responses captured by the WDS software.

The purpose of this experiment was to investigate the presence of any data pattern that can be recognised by the analysis of the captured dataset. In Figure 15, the red marks indicate the values that correspond to the voltages of each node. The RSSI signal is related to the RF signal power of the message received by the radio transceiver, and it is dependent of the radio power and the distance between the source and the destination nodes. The sink node (raspberry kit) and the sensor nodes (ARM kits) use the same transceiver radio (MRF24J40), and they are equidistant from the Zena adapter. Thus, the RSSI signals of different frames can present the same or almost the same values. On the other hand, if the sensor node is further from the Zena wireless adapter, the RSSI signal of its frame will be different from the RSSI signal of the sink’s frame. After one hour of communication among nodes, the ZENA wireless adapter captured 100 response packets. For each packet, we extracted the encrypted field corresponding to the voltage value, as shown in Figure 16.

From the analysis of the sample distribution and two estimation techniques by both points and intervals, we are not able to find any data pattern. Hence, even if a spy node captures the data communicated through the network, it would be difficult to decipher the values stored in the message fields.

## 5. Conclusions

We propose a middleware that runs on the sink node of a ZigBee-based WSN to integrate legacy electrical equipment into a smart grid that uses standard communication protocols, such as DNP3, IEC 61850, or Modbus. This middleware allows, as its main feature, the translation of messages communicated between the PSCC and electrical equipment in the smart grid.

After surveying different infrastructures, middleware programs, and converters to integrate the smart-grid components using WSNs, we found that this is the first work that proposes an infrastructure to integrate a WSN into a smart grid operating with three different protocols and to satisfy some requirements for smart-grid applications.

In fact, we used several strategies to meet these requirements. As a result, one of the proposed strategy was able to meet the response time requirement of the IEC61850 protocol for real-time monitoring. Moreover, we implemented strategies to improve the security in the WSN communication and to automatically configure and include new sensor nodes to the network. The results from various experiments verified that these strategies are effective in a WSN composed of a few sensor nodes.

In future work, we will evaluate WSN characteristics such as scalability, response time, and reliability from simulations using the Castalia software to improve the proposed infrastructure. Moreover, we will make an agreement with a power utility to install a neighbourhood area network (NAN) with several electronic power meters to evaluate the response time, reliability in communication, and scalability in a real deployment. Analytical tests will be carried out in a smart metering real scenario to evaluate mainly the response time and reliability characteristics in WSN communication based on the number of sensor nodes, their distributions and distances.
